# Characteristics and risk factors for mortality among hospitalized patients with pulmonary tuberculosis in Shizuoka Prefecture in 2009–2020

**DOI:** 10.1186/s12890-026-04521-7

**Published:** 2026-07-20

**Authors:** Kei Kanata, Toshihiro Shirai, Yutaro Kishimoto, Taisuke Akamatsu, Yutaro Ito, Koshiro Ichijo, Masahiro Uehara

**Affiliations:** 1 Department of Respiratory Medicine, Shimada General Medical Center, 1200-5 Noda, Shizuoka, Shizuoka Prefecture 427-8502 Japan; 2 Department of Respiratory Medicine, Shizuoka General Hospital, Shizuoka, Japan

**Keywords:** Tuberculosis, Low-burden country, Immigrants, Prognostic factors, Elderly patients

## Abstract

**Background:**

Japan had long been a medium-burden country for tuberculosis (TB) but became a low-burden country in 2021. Shizuoka Prefecture is geographically located in Central Japan, and the incidence rate of TB is also at the average level in Japan.

This study aimed to understand TB patients' characteristics and risk factors by analyzing the data from 2009 to 2020 in Shizuoka Prefecture, a representative sample for Japan.

**Methods:**

We retrospectively collected 1,132 patients with smear-positive pulmonary TB who were admitted to Shimada General Medical Center or Shizuoka General Hospital in the central area of Shizuoka Prefecture from 2009 to 2020. Patients were divided into alive and dead (i.e., all-cause in-hospital death during anti-tuberculosis treatment) groups, and clinical parameters were compared between the two groups.

**Results:**

The median age of all patients was 80. The number of patients decreased from 2009 to 2020. Moreover, the number of patients over 70 gradually reduced, which was similar for all patients. Immigrant TB patients were 5% of all patients, which was very small compared to the total number of patients. Almost all were from Southeast Asian countries. In multivariate analysis, older age, low body mass index, respiratory failure, low serum albumin, lymphocyte, high serum creatinine levels, and neutrophil were independent prognostic factors.

**Conclusions:**

The decline in TB among elderly patients, who comprise most of the population, might be one reason why Japan has become a low-burden country. Although the rate of immigrants is minimal, caution is needed about the increase, especially from Southeast Asian countries. There is a need for a TB medical system that can deal with complications and malnutrition, which are risk factors for death.

## Background

*Tuberculosis* (TB) is an airborne disease that infects 10.6 million people worldwide. Before the emergence of the novel coronavirus disease (COVID-19), TB was the leading cause of death from a single pathogen worldwide. Even now, 1.3 million people die in 2022 [1]. The incidence of TB is exceptionally high in developing countries and tends to be low in developed countries. In the 2021 analysis, the country with the highest incidence of TB is the Philippines (incidence rate 650), followed by the Kingdom of Lesotho (614), which is surrounded by the Republic of South Africa (513). The lowest incidence rate is in the United States (2.6) [2].

Japan had been a medium-burden country for TB for a long time but became a low-burden country in 2021 [3]. Hagiya et al. reported that TB contact investigations routinely performed by public health centers, as government support, could have contributed to the decrease in TB prevalence in Japan [4]. The Japan Anti-Tuberculosis Association suggested that a decrease in the incidence rate was detected after the TB emergency was declared in 1999, and this declaration may have had some effect [5]. But the direct cause remains unclear.

Imported TB from immigrants is also a challenge in eliminating TB because Japan has an increasing number of immigrants, mainly from Asia. The proportion of immigrants among all TB cases in Japan has steadily increased from 2.4% in 2000 to 10.7% in 2019 [6].

**Fig. 1:**
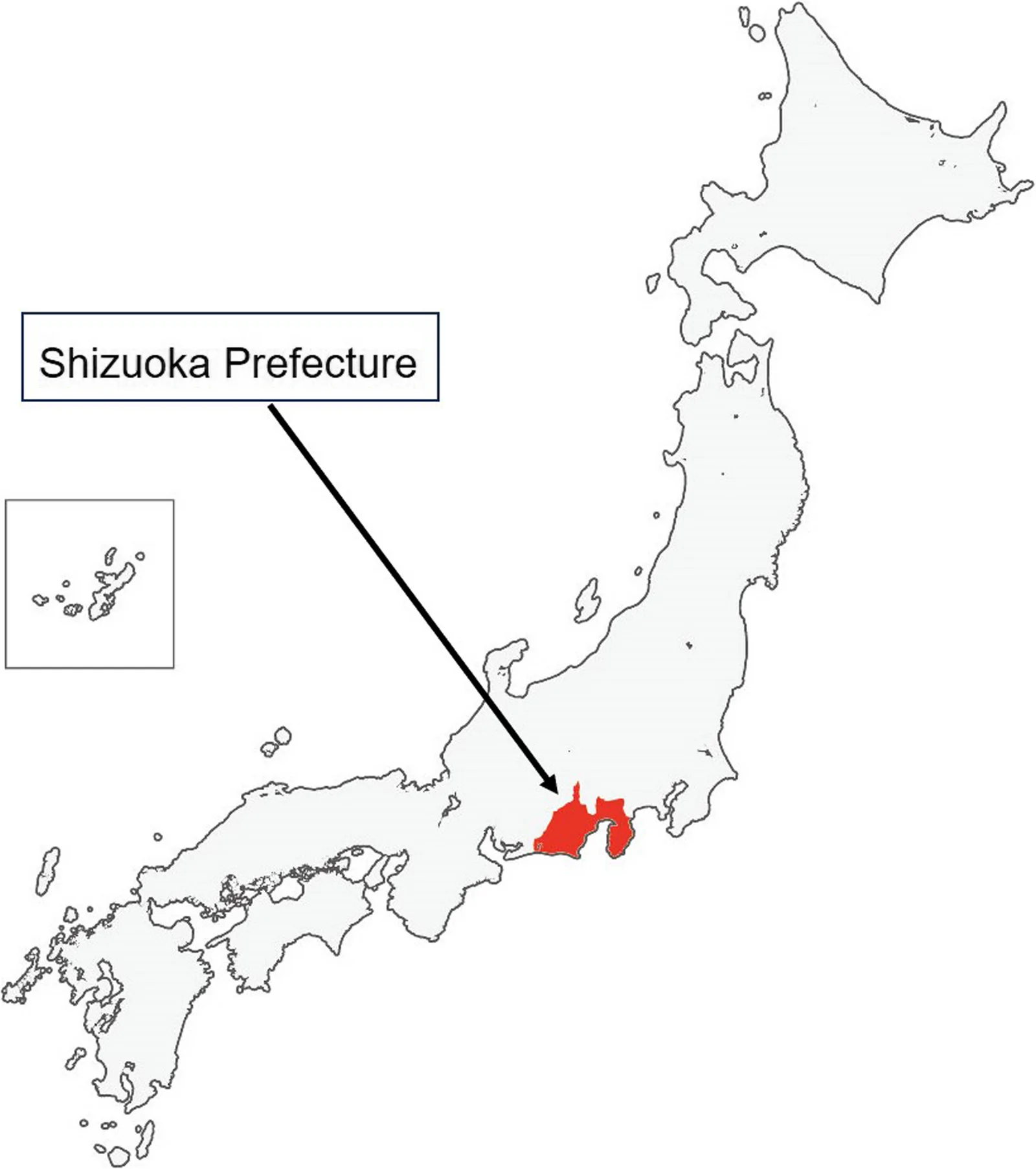
Location of Shizuoka Prefecture. It is geographically located in the center of Japan

Shizuoka Prefecture is geographically located in Central Japan (Fig. 1), and the incidence rate of TB is also at the average level in Japan [7]. Therefore, this study aimed to understand TB patients’ characteristics and risk factors for mortality by analyzing the data from 2009 to 2020 in Shizuoka Prefecture, a representative sample of Japan.

## Methods

### Study design and data collection

We applied for permission to use data from Shimada General Medical Center and Shizuoka General Hospital and received approval from the institutional review board (R 6 − 5). The board waived patient approval or informed consent because the study was a retrospective review of patient records. We retrospectively collected 1,132 patients with smear-positive pulmonary TB who were admitted to Shimada General Medical Center or Shizuoka General Hospital in the central area of Shizuoka Prefecture from 2009 to 2020. The inclusion criteria for this study were as follows: pulmonary TB as a definitive diagnosis. Acid-fast sputum smears were positive; subsequently, Mycobacterium tuberculosis was isolated by culture in all patients. We excluded cases with no definitive diagnosis of TB, no description of outcome, or smear-negative patients.

Medical records were used to collect clinical and demographic information and peripheral blood tests. Sputum smears were obtained before treatment and classified according to the Gaffky scale. Patients were divided into alive group, who were discharged alive after TB treatment during hospitalization, and the dead group, who died in-hospital due to all-cause death during TB treatment, and clinical parameters were compared between the two groups. All patients received standard anti-tuberculosis treatment during hospitalization in accordance with the Japanese national guidelines.

### Statistical analysis

Categorical variables are summarized as frequencies and proportions, and continuous variables are expressed as medians and interquartile ranges. We used the Mann-Whitney U and Fisher’s exact test for nonparametric data to evaluate the difference between alive and dead groups. Variables contributing to death were subjected to multivariate analysis by logistic regression after univariate analysis. All analyses were performed with R version 3.6.3 (R Foundation for Statistical Computing, Vienna, Austria). A two-tailed p-value < 0.05 was considered significant.

## Results

### Characteristics of the subjects

The clinical characteristics of the study population on admission are shown in Table 1. The median age of all patients was 80, and male patients were dominant. The median body mass index (BMI) was 18.6. Respiratory failure, corticosteroid use, and malignant disease were the main primary pre-existing diseases. Slightly low albumin and hemoglobin were observed in laboratory data. Dead patients were older, had lower BMI, more immigrants, more extended hospital stays, more miliary tuberculosis, respiratory failure, corticosteroid users, chronic renal failure, lower levels of serum albumin, lymphocytes, hemoglobin, and higher levels of serum creatinine, AST, neutrophils, Gaffky scale than discharged alive patients.

**Table 1:** Characteristics of the patients

Characteristics	All patients (*n* = 1132)	Discharged alive group (*n* = 906)	Dead group (*n* = 226)	*P* value
Age, yr	80 (68–87)	78 (63–85)	87 (80–90)	< 0.001
Sex, male	709 (63)	563 (62)	146 (65)	0.49
BMI, kg/m^2^	18.6 (16.5–20.9)	19.0 (17.1–21.2)	16.8 (15.2–18.7)	< 0.001
Immigrant patients	58 (5)	58 (5)	0 (0)	< 0.001
Length of hospital stays, d	64 (46–99)	69 (51–104)	29 (12–64)	< 0.001
Comorbidity				
Miliary tuberculosis	60 (5)	35 (4)	25 (11)	< 0.001
Respiratory failure	352 (31)	189 (21)	163 (72)	< 0.001
Corticosteroid user	176 (16)	111 (12)	65 (23)	< 0.001
Malignant disease	109 (10)	86 (9)	23 (10)	0.76
Cardiac failure	59 (5)	40 (4)	19 (8)	0.02
Diabetes	49 (4)	41 (5)	8 (4)	0.52
COPD	22 (2)	14 (2)	8 (4)	0.08
Cerebral infarction	24 (2)	17 (2)	7 (3)	0.28
Liver cirrhosis/hepatitis	12 (1)	9 (1)	3 (1)	0.67
Bronchial asthma	11 (1)	7 (1)	4 (2)	0.21
Chronic renal failure	9 (1)	3 (0)	6 (3)	0.002
Interstitial pneumonia	8 (1)	5 (1)	3 (1)	0.25
Laboratory data				
Alb, g/dL	2.90 (2.35–3.20)	3.10 (2.50–3.60)	2.20 (1.80–2.60)	< 0.001
Crt, mg/dL	0.74 (0.60–0.96)	0.70 (0.60–0.93)	0.87 (0.60-1.00)	< 0.001
AST, IU/L	25 (20–34)	22 (17–31)	31 (21–48)	< 0.001
ALT, IU/L	16 (11–23)	15 (11–23)	18 (11–31)	0.71
WBC, cells/µL	6650 (5275–8350)	6900 (5500–8600)	7100 (4900–9500)	0.12
Neutrophil, cells/µL	5290 (4020–7038)	5225 (4015–6777)	5929 (4111–8569)	< 0.001
Lymphocyte, cells/µL	852 (527–1236)	953 (624–1320)	476 (298–735)	< 0.001
Hemoglobin, g/dL	11.4 (10.3–13.0)	11.8 (10.5–13.2)	10.7 (9.2–12.1)	< 0.001
Gaffky scale	5 (2–8)	5 (2–8)	6 (2–9)	0.004
Drug resistance (MDR/XDR)	14 (1)	12 (1)	2 (1)	0.68

Data are shown as median (interquartile range) or frequency (percentage)

*Abbreviations*: *Alb* Albumin, *ALT* Alanine aminotransferase, *AST* Aspartate aminotransferase, *BMI* Body mass index, *COPD* Chronic obstructive pulmonary disease, *Crt* Creatinine, *MDR* Multi-drug-resistant, *WBC* White blood cell, *XDR* Extensively drug-resistant

### Changes in the patient number by background over 12 years

**Fig. 2:**
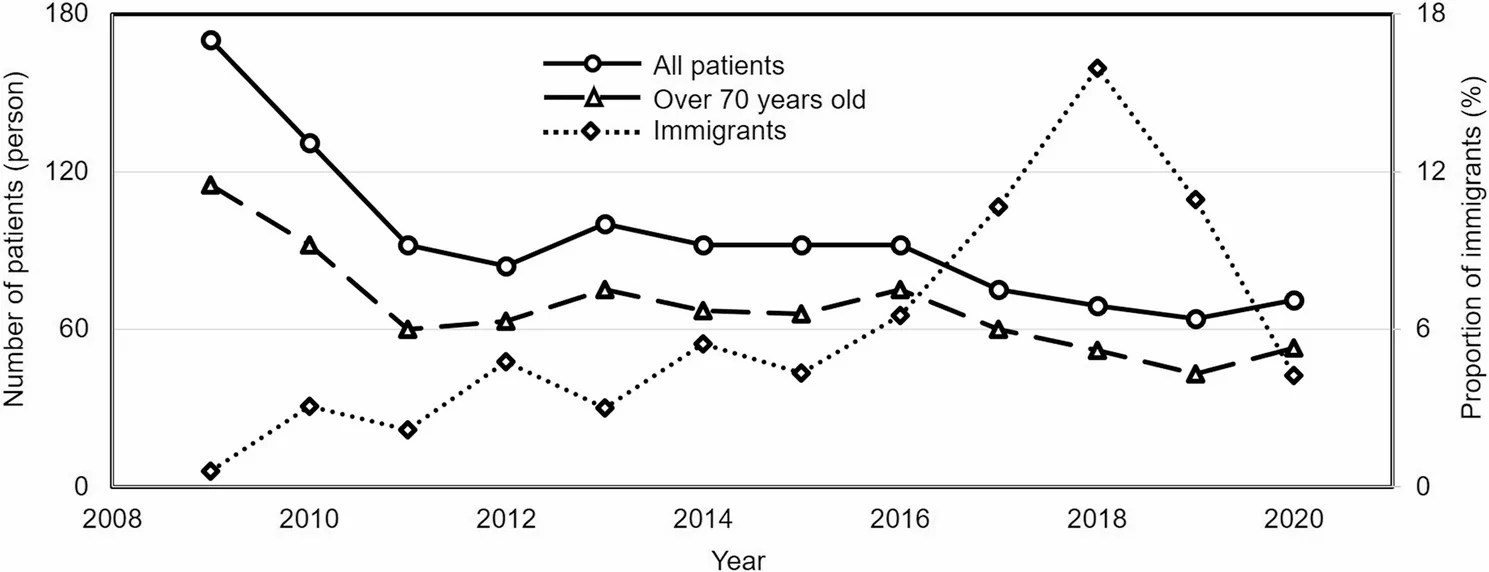
Changes in the patient number and the proportion of immigrants. The number of smear-positive pulmonary TB patients decreased from 2009 to 2020. The number of patients over 70 years old also gradually reduced. The proportion of immigrants gradually increased until 2018 and declined after that

The number of all patients decreased from 2009 to 2020 (Fig. 2). The number of patients over 70 years old also gradually reduced. The proportion of immigrant patients each year is shown in Fig. 2. The ratio of immigrants gradually increased until 2018 and decreased after that.

### Characteristics of immigrant TB patients

**Fig. 3:**
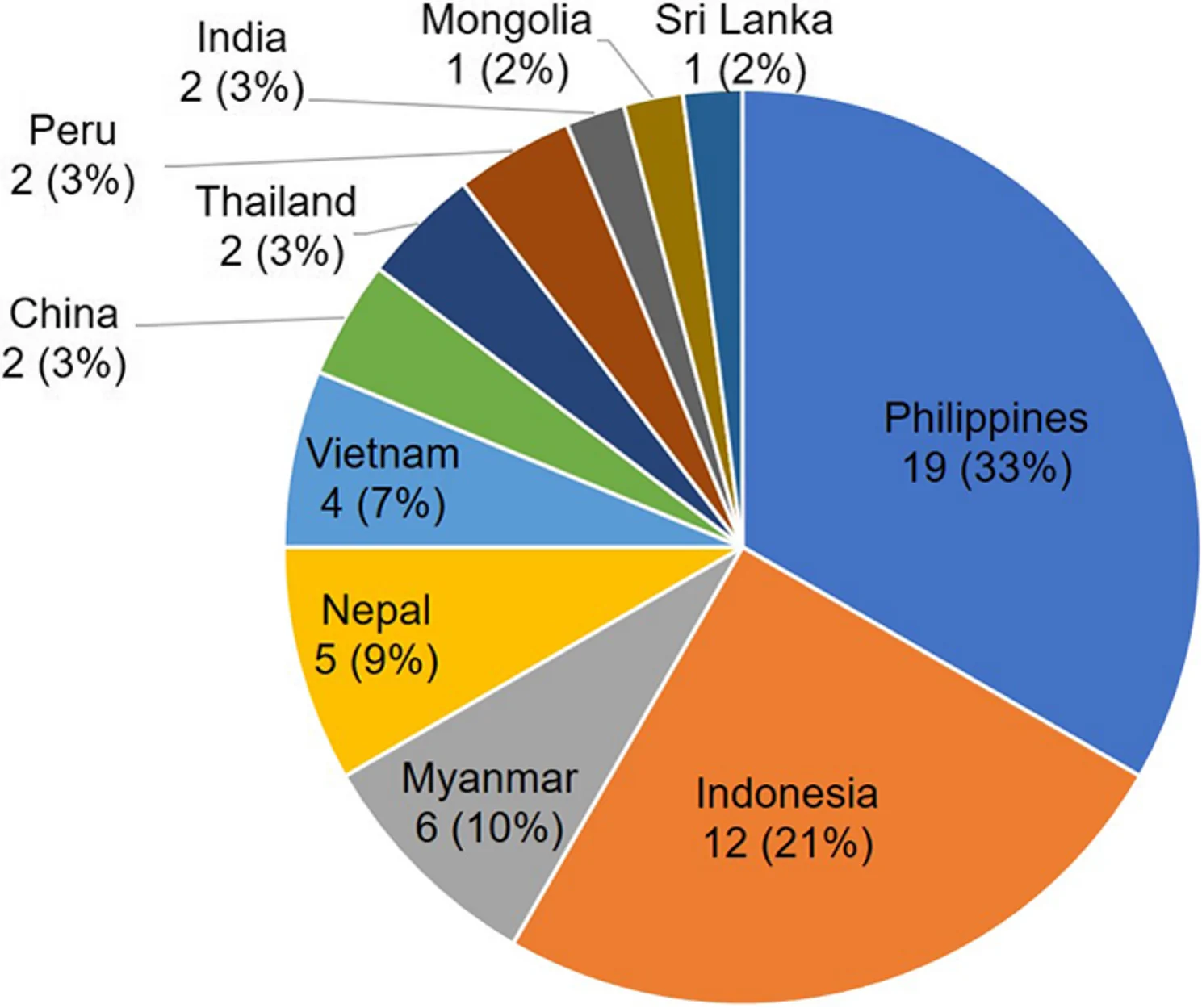
The nationality of immigrants. They corresponded to 5% of all patients, most from Southeast Asia, including the Philippines, Indonesia, and Myanmar. There were few patients from South American countries

**Fig. 4:**
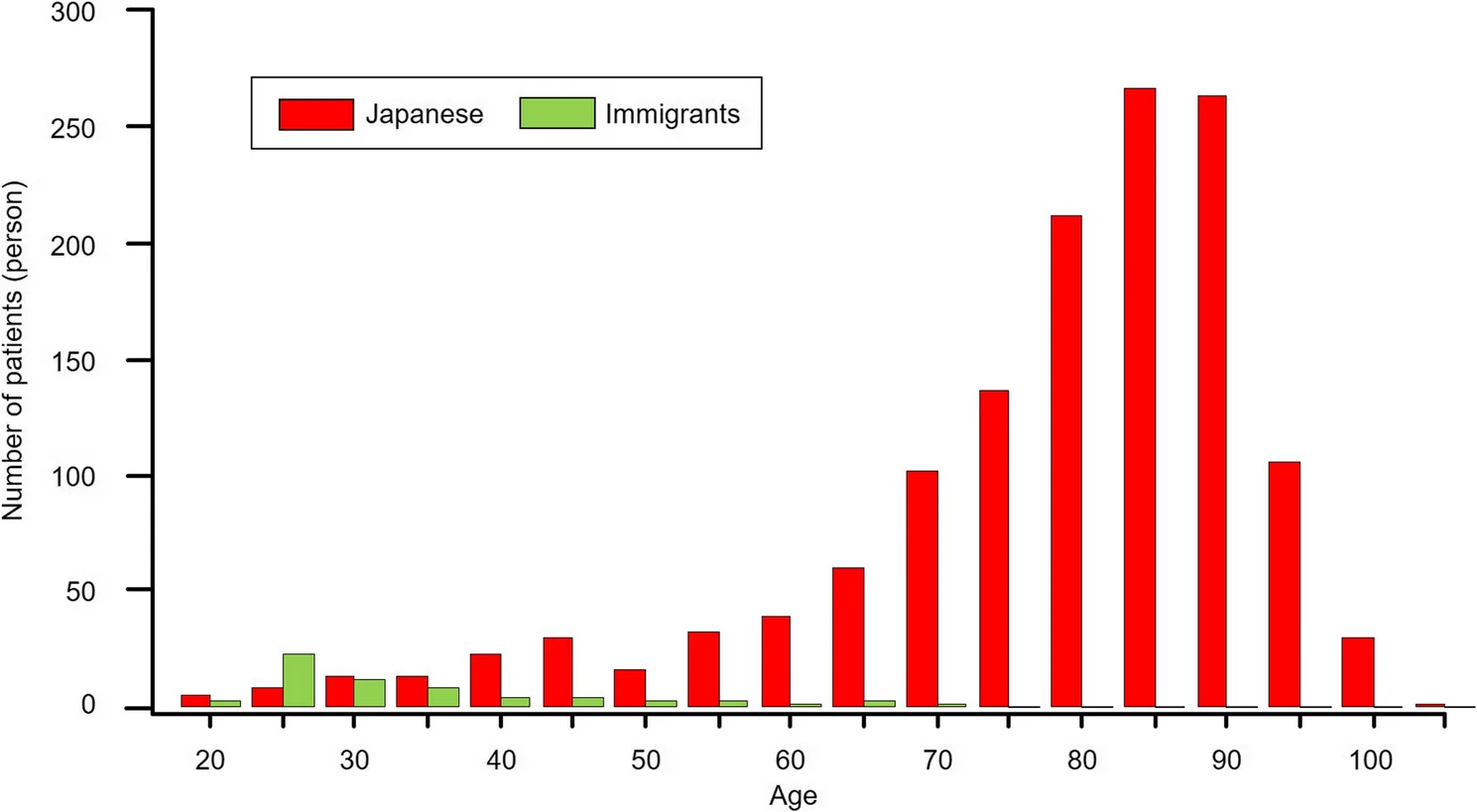
The age distribution of hospitalized smear-positive patients. Immigrants were younger than Japanese patients

Immigrant TB patients were 5% of all patients, which was very small compared to the total number of patients. Almost all were from Southeast Asian countries, including the Philippines, Indonesia, and Myanmar (Fig. 3). There were few patients from South American countries. Immigrant patients were younger than Japanese patients (Fig. 4), and all were discharged alive (Table 1).

### Multiple logistic regression analysis for factors associated with dead patients

As shown in univariate analysis (Table 2), older age, low BMI, miliary TB, respiratory failure, corticosteroid use, chronic renal failure, low levels of serum albumin, lymphocyte, hemoglobin, high levels of serum creatinine, AST, and neutrophils were independent prognostic factors. In multivariate analysis, older age, low BMI, respiratory failure, low serum albumin, lymphocyte, high serum creatinine levels, and neutrophil were independent prognostic factors.

**Table 2:** Univariate and multivariate analysis of factors contributing to death from hospital discharge

	Univariate analysis			Multivariate analysis		
	Odds ratio	95% CI	*P* value	Odds ratio	95% CI	*P* value
Age, yr	7.72	4.79–12.4	< 0.001	1.11	1.08–1.14	< 0.001
Sex, male	1.11	0.82–1.51	0.494			
BMI, kg/m^2^	0.30	0.21–0.42	< 0.001	0.87	0.80–0.95	0.001
Comorbidity						
Miliary tuberculosis	3.05	1.81–5.16	< 0.001	0.91	0.41–2.03	0.818
Respiratory failure	9.75	7.00-13.6	< 0.001	6.41	4.05–10.2	< 0.001
Corticosteroid user	3.08	2.17–4.37	< 0.001	1.45	0.85–2.50	0.176
Malignant disease	1.08	0.67–1.75	0.755			
Cardiac failure	1.60	0.91–2.83	0.025			
Diabetes	0.77	0.36–1.68	0.515			
COPD	2.34	0.97–5.64	0.094			
Cerebral infarction	1.67	0.68–4.08	0.204			
Liver cirrhosis/hepatitis	1.34	0.36–4.99	0.661			
Bronchial asthma	2.31	0.67–7.97	0.172			
Chronic renal failure	8.21	2.04–33.1	0.002	1.23	0.06–23.6	0.890
Interstitial pneumonia	2.42	0.58–10.2	0.213			
Laboratory data						
Alb, g/dL	0.08	0.05–0.12	< 0.001	0.25	0.16–0.40	< 0.001
Crt, mg/dL	2.43	1.80–3.27	< 0.001	1.45	1.17–1.78	< 0.001
AST, IU/L	3.36	2.46–4.59	< 0.001	1.01	1.00-1.01	0.153
ALT, IU/L	1.01	0.96–1.06	0.710			
WBC, cells/µL	1.01	0.99–1.01	0.110			
Neutrophil, cells/µL	1.82	1.36–2.44	< 0.001	1.22	1.09–1.36	< 0.001
Lymphocyte, cells/µL	0.16	0.11–0.24	< 0.001	1.26	1.08–1.46	0.003
Hemoglobin, g/dL	0.43	0.32–0.59	< 0.001	1.04	0.91–1.18	0.57
Gaffky scale	1.07	1.02–1.11	0.004	1.05	0.97–1.12	0.234

*Abbreviations*: *Alb* Albumin, *ALT* Alanine aminotransferase, *AST* Aspartate aminotransferase, *BMI* Body mass index, *COPD* Chronic obstructive pulmonary disease, *Crt* Creatinine, *WBC* White blood cell

## Discussion

This study shows TB patients’ characteristics and risk factors in Shizuoka Prefecture, a representative sample of Japan, helping us understand the subjects’ characteristics and changes in the patient number by background and factors associated with dead patients.

Shizuoka Prefecture is one of the prefectures with an aging population, which is the same trend in Japan. The results show that the median age of TB patients in Shizuoka Prefecture was very old. Moreover, they are gradually getting older compared with a few decades ago [3]. The poor outcomes in patients who had low BMI and complicated diseases were thought to be due to a decline in nutritional status with age.

This study also indicated that TB is approximately 1.7 times more common in males than in females. Previous research examining the incidence of TB in the aspect of sex revealed that men are more likely to be diagnosed with TB than women, with a male-to-female ratio of 1.6:1 globally [8]. This sex gap has been proposed to be explained by biological differences in disease and disease presentation and different access to health care, specifically in developing countries [9–11]. Additionally, men are more likely to report risk factors associated with TB exposure [9, 11–14].

Our analysis also found that the number of patients over 70, who comprise most of the population, is decreasing. In Japan, TB patients have decreased due to the widespread use of BCG vaccines and antibiotics to prevent TB, improved sanitary conditions, and more substantial guidance on medication administration by public health nurses. Experts mention that the declining TB cases could be partly explained by a drop in the number of people seeing a doctor and undergoing scaled-back health screenings by public health centers amid the coronavirus pandemic [15]. For these reasons, TB among elderly patients, who comprise most of the population, has declined, which may be one reason why Japan has become a low-burden country.

In our analysis, almost all immigrants of central Shizuoka Prefecture were from Southeast Asia, and there were few patients from South American countries. Looking at the western Shizuoka Prefecture, the number of TB cases among immigrants from South America is most prevalent. This is because Hamamatsu City, the largest city in the west of Shizuoka prefecture, has the transportation equipment industry, such as automobiles and motorcycles, and immigrants from South America, including Brazil, tend to work in these companies [16]. This study found differences in immigrants’ countries of origin in western and central Shizuoka Prefecture.

Immigrant patients were younger than Japanese patients, which means they had good nutrition and few complicated diseases, which led to all of the immigrants being discharged alive. Though immigrants are very small now, the number of immigrants may increase in the future. Thus, it must be necessary to prepare for immigration outbreaks.

Other novel findings of our study were that low BMI, respiratory failure, low serum albumin levels, lymphocyte, and high serum creatinine levels were independent prognostic factors for death. Osawa et al. reported a clinically applicable mortality risk prediction system for pulmonary TB. The disease severity score (named the AHL score) included daily living, hypoxemia, and lymphocytes (< 720/µL). This score showed good discrimination of mortality risk [17]. However, there were no reports about these comprehensive data about prognostic factors by clinical and demographic information and peripheral blood tests. In Japan, the number of TB patients is getting older, and complicated diseases will increase compared with the past. Therefore, it is necessary to create a medical system to deal with this problem.

This study has several limitations. First, the analysis was limited to smear-positive TB patients and conducted at only two hospitals within a single prefecture. This limitation may affect the generalizability of the findings and might lead to selection bias. Second, we were unable to evaluate TB-specific mortality, limiting our ability to provide insights into disease-specific outcomes. Third, important variables such as socioeconomic status—known to influence TB prognosis—were not included, potentially affecting the comprehensiveness of the analysis. Future studies, including a more diverse patient population, broader geographic settings, and additional prognostic variables, are needed to validate and extend these findings.

## Conclusions

The analysis of two hospitals in Shizuoka Prefecture suggests that the decline in TB among elderly patients, who comprise most of the population, might be one reason why Japan has become a low-burden country. Although immigrants are very small now, caution about the increase in immigrants from Southeast Asian countries is needed. In addition to patients’ age, it is expected that the number of patients with complications and malnutrition, which are factors for poor prognosis, will increase. There is a need for a TB medical system that can deal with these complex underlying diseases.

## Data Availability

The data that support the findings of this study are available from the institutional review board in Shimada General Medical Center but restrictions apply to the availability of these data, which were used under license for the current study, and so are not publicly available. Data are however available from the authors upon reasonable request and with permission of the institutional review board in Shimada General Medical Center.

## Abbreviations

TB: Tuberculosis
COVID-19: Coronavirus disease 2019
BMI: Body mass index
AST: Aspartate aminotransferase
BCG: Bacille de Calmette et Guérin
